# Aging and hypertension in kidney function decline: A 10 year population-based study

**DOI:** 10.3389/fcvm.2022.1035313

**Published:** 2022-10-06

**Authors:** David A. Jaques, Peter Vollenweider, Murielle Bochud, Belen Ponte

**Affiliations:** ^1^Division of Nephrology, Department of Medicine, Geneva University Hospitals, Geneva, Switzerland; ^2^Department of Internal Medicine, University Hospital of Lausanne, Lausanne, Switzerland; ^3^Department of Epidemiology and Health Systems, University Center of General Medicine and Public Health, Lausanne, Switzerland

**Keywords:** aging, hypertension, kidney function, eGFR, population

## Abstract

**Background:**

Aging is associated with a physiological decline in kidney function (KFD). In this study, we aimed to describe the impact of age on the rate of KFD and its interplay with risk factors for chronic kidney disease (CKD), considering mainly hypertension (HT), in the general population.

**Materials and methods:**

Participants of European descent, aged 35–75, were recruited from a populational cohort in Lausanne, Switzerland. Participants with a 10 year follow-up were selected. KFD was defined as the difference in estimated glomerular filtration rate (eGFR) between baseline and follow-up, divided by the observation period. Multivariate linear regressions were used with KFD as the outcome and age as the main predictor. HT was tested as a modifying factor.

**Results:**

We included 4,163 participants with mean age 52.2 ± 10.4, 44.7% men, 31.9% HT, and 5.0% diabetics. Mean baseline eGFR was 85.9 ± 14.6 ml/min/1.73 m^2^. Mean KFD was –0.49 ± 1.08 ml/min/1.73 m^2^ per year with 70% of participants decreasing their eGFR during follow-up. The relationship between age and KFD was non-linear and age was divided in tertiles. Old participants had faster rates of KFD as compared to young and middle-age participants (*p* < 0.001). A significant interaction was found between age and HT on KFD prediction (*p* < 0.001). In HT participants, KFD was significantly different across tertiles of age (*p* < 0.001). On contrary, KFD was not different across tertiles of age in non-HT participants.

**Conclusion:**

A physiological KFD is present over time in the general population. Age contributes non-linearly to the rate of this decline with older subjects declining the fastest. The presence of HT is a major contributing factor in this setting as KFD worsened with age only in hypertensive participants. Thus, HT represents an important pathological factor aggravating the age-related physiological decline in eGFR in the general population.

## Introduction

Chronic kidney disease (CKD) is a leading cause of morbidity and mortality worldwide, mainly attributed to cardiovascular disease (1). In the United States, 37 million people and 15% of adults are estimated to have CKD (2). In Europe, there is a large variation of CKD prevalence, ranging from 3.3 to 17.3% across countries (3). In the adult population of Switzerland its prevalence is 10% but increases with diabetes, hypertension (HT), and aging (4). Moreover, the prevalence of CKD defined by a single threshold of glomerular filtration rate (eGFR) < 60 ml/min/1.73 m^2^ is growing in elderly with more than half of adults over 70 classified as having CKD (5). However, as there is a physiological decline in kidney function with aging, whether a mild decrease in eGFR in the elderly actually represents a pathological state is debated. Since 1950, studies have shown a yearly decrease of around 1 ml/min of eGFR from the age of 40 (6–8). Significant discrepancies in the rate of kidney function decline (KFD) exist, however, in cross-sectional and longitudinal studies (9). In addition, several studies reported steeper KFD but represented secondary analyses from prior randomized controlled trials in patients with CKD or population-based studies in elderly (10). Globally, few longitudinal population-based studies focusing on KFD have been conducted (11–15). Two of them described the longitudinal effect of age on KFD in large communities from Israel and Japan, respectively (14, 15). The other studies focused on older adults (11–13). However, none of these previous reports accounted for albuminuria, a major predictor of KFD (16, 17). Thus, while demographical, clinical, and biological factors associated with KFD and incident CKD have been previously reported, the longitudinal impact of age and its interplay with those factors is unclear (18–20). KFD is associated with mortality as well as incidence of end-stage kidney disease (ESKD) and has thus been proposed as a surrogate endpoint of adverse kidney event (21–23). Consequently, the general aim of the present study was to comprehensively describe the effect of age on KFD over a long-term period in a population-based cohort. Specifically, we wished to confirm an age-related decline in kidney function in a general population, test the hypothesis that this decline is not uniform across age categories and describe the interplay of age with other factors associated with KFD, mainly HT. Finally, we wanted to determine whether aging is associated with accelerated KFD or the incidence of CKD.

## Materials and methods

### Participants

We used the CoLaus cohort for the present study. CoLaus is a prospective cohort from the general population, including at baseline 6,188 participants from European descent, aged 35–75. The main purpose of this cohort was to study epidemiological and genetic determinants of cardiovascular risk factors and kidney function. Selection of participants and baseline data have been described in detail previously (24). Briefly, initial recruitment took place in Lausanne, a Swiss city of 117,161 inhabitants. In 2003, the population register from Lausanne provided a complete list of the residents aged 35–75 (*N* = 56,694). A simple, non-stratified random sample of 35% of this source population was drawn. Selected subjects were contacted by letter and presented with the main purpose of the cohort. The recruitment began in June 2003 and ended in May 2006. A total of 8,121 adults agreed to take part, representing 41% of the initial population. Inclusion criteria were: (a) aged 35–75, (b) written informed consent, and (c) Caucasian origin. Caucasian origin was defined clinically as having both parents and grandparents born in a restricted list of countries. There were no exclusion criteria. At baseline, 6,184 Caucasians adults were included. All CoLaus participants were invited to 5 and 10 year follow-ups. Only participants having both baseline and 10 year follow-up data were included in the present analysis.

### Ethics

This study involving human participants was reviewed and approved by the local ethics committee “Commission cantonale d’éthique de la recherche sur l’être humain” (CER-VD: VD-16/03; VD-33/09, and VD-26/14) and was conducted in accordance with the declaration of Helsinki.

### Variables

Variables collection was identical at baseline and 10 year follow-up. Participants were asked to attend the outpatient clinic at Centre Hospitalier Universitaire Vaudois (CHUV), Lausanne, Switzerland, in the morning after an overnight fast. Venous blood samples were drawn and random morning single urine specimens were collected. During a face-to-face meeting with trained field interviewers, participants were requested to fill standardized questionnaires. The questionnaire collected demographic data, socioeconomic status (education, occupation), lifestyle factors (physical activity, alcohol intake, and smoking), and current medication. Personal and family history on cardiovascular risk factors or events was also recorded. During the clinical visit, blood pressure (BP) and heart rate were measured three times in a sitting position using an automatic oscillometric device. Height and weight were measured using standardized scales. BMI was calculated and expressed as kg/m^2^. Standard laboratory analyses were performed at the CHUV clinical laboratory on fresh samples. In blood, were measured: creatinine, markers of diabetes and insulin resistance, lipids, liver tests, inflammatory markers, uric acid, albumin, and blood count. In urine, were measured: creatinine, albumine, and electrolytes. IDMS-traceable Jaffe kinetic compensated method was used to measure creatinine in blood and urine (Roche Diagnostics, Switzerland, intra- and inter-batch CV 2.9–0.7%). Glucose was measured by Glucose Deshydrogenase (Roche Diagnostics, Switzerland, intra- and inter-batch CV 2.1–1.0%). Serum uric acid was measured using uricase-PAP (inter- and intra-batch CV 1.0–0.5%). Serum ultrasensitive C-reactive protein (CRP) was measured by Immunoasay and latex HS (Agilent 1100, Switzerland, inter- and intra-batch CV 4.6–1.3%). Quantitative immuno-nephelometry was used to measure albumin in urine.

### Definitions

We have previously described baseline kidney function in the CoLaus cohort (25). Race-adjusted 2009 creatinine CKD-EPI equation was used to calculate eGFR in ml/min/1.73 m^2^ (26). Urinary albumin-to-creatinine ratio (ACR) was calculated to estimate albuminuria in mg/g. Presence of albuminuria was defined as ACR > 30 mg/g. Presence of CKD was defined solely by eGFR < 60ml/min/1.73 m^2^. Decline in eGFR was defined as a decrease in eGFR from baseline to follow-up at 10 years. KFD was defined as the difference in eGFR between baseline and follow-up at 10 years, divided by the follow-up period. The follow-up period was defined as the difference between the date of the first observation and the date of the last examination in years. KFD was thus expressed as ml/min/1.73 m^2^ per year. Rapid KFD was defined as an annual KFD ≥ 3 ml/min/1.73 m^2^ (27). HT was defined as the use of anti-hypertensive drug or a mean systolic/diastolic BP ≥ 140/90 mmHg on office measurement (25). Diabetes was defined as the use of antidiabetic drug (oral or injectable) or a fasting glucose ≥7 mmol/L. Dyslipidaemia was defined as the use of lipid lowering drug. Smoking was defined as currently smoking vs. non- or former-smoking. Education level was separated in three categories: high (tertiary education), middle (upper secondary education or post-secondary non-tertiary education), and low (lower or no secondary education). Tertiles of age were created to obtain the same number of patients in three categories.

### Statistics

In descriptive analysis, continuous variables were expressed as mean ± standard deviation (SD) or median (interquartile range) according to distribution. Variables were compared between two groups using *T*-test (or Wilcoxon test) and Chi2 for continuous and categorical variables respectively. Variables were compared between three groups using ANOVA (or Kruskal-Wallis) and Chi2 for continuous and categorical variables respectively. For the main analyses, multivariate linear regression models were used with KFD as the outcome and age as the main predictor. In addition to unadjusted analysis, multivariate models were constructed. In model 1, the following covariates were considered: gender, education level, dyslipidaemia, CRP, and uric acid. In model 2, diabetes, HT, BMI, and CKD were added as covariates. In model 3, ACR was added as covariate. Those covariates were selected based on prior scientific knowledge of their effect on kidney function as well as individual significant association with both KFD and age in exploratory analyses (see section “Results”) (18). Variables were log-transformed when necessary (CRP, ACR). A graphical representation of KFD as a function of age showed a non-linear relationship (see section “Results”) and departure from the line was confirmed in regression models. As such, age was considered a three-level categorical variable (tertiles) in linear models in order to satisfy statistical assumptions. Results are expressed as absolute β coefficients, 95% confidence intervals (95% CI), and associated *p*-values for each individual age tertiles. Global differences across tertiles as well as the relative effect of each age tertile in relation to one another were also tested. Interaction effect between age and hypertension was tested comparing models with interaction term (age × HT) to models without (age + HT). Interaction was considered significant when *p*-value for likelihood ratio test (LRT) comparing both models was <0.05. In addition to HT, gender was also considered as a potential interacting term based on prior scientific knowledge (28–30). Of note, only 63 patients (10, 19, and 34 in each age tertile) had diabetes without HT, thus compromising the analysis of diabetes alone. Therefore we could not test interaction with diabetes. Sensitivity analyses were conducted with an alternative definition of HT and with adjustment for various anti-hypertensive medications. In secondary analyses, multivariate logistic regression models were used with rapid KFD or incident CKD as the outcomes and age as the main predictor. Only patients without CKD at baseline were included in incident CKD analyses. Models specifications were identical to linear regression (see above). Results are expressed as odds ratio (OR), 95% CI, and associated *p*-values. Gender and HT were also considered as potential interacting terms.

## Results

### Baseline characteristics

**FIGURE 1:**
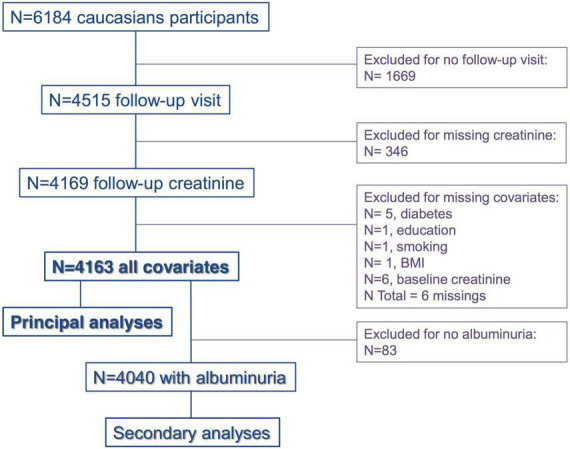
Study flowchart. BMI, body mass index.

From the 6,184 initial participants, 4,515 (73.0%) had a follow-up visit at 10 years and 4,169 (67.4%) had available creatinine measurement. Of those, six had missing values on considered covariates and 4,163 participants were thus included in the main analyses. Study flow chart is provided in Figure 1. Regarding participants who did not have a follow-up visit at 10 years (*N* = 1,669), 179 (10.7%) died, and 861 (51.6%) were still alive. No information on the status of the 629 (37.7%) remaining subjects was available. Characteristics of the 4,163 included participants as compared to the 2,021 excluded subjects are described in Supplementary Table 1. Compared to included patients, the excluded ones were older, more frequently men and smoker, had lower education, higher BMI and a higher prevalence of HT, diabetes, dyslipidaemia, CKD, and albuminuria. The mean age of the 4,163 included participants was 52.2 ± 10.4 with 44.7% men. Mean BMI was 25.5 ± 4.3 kg/m^2^ with 5.0% diabetics, 24.8% smokers, and 9.9% with dyslipidaemia. In total, 1,327 patients (31.8%) were hypertensive according to our definition (see section “Materials and methods”). Among those, 694 (16.6%) had systolic/diastolic BP ≥ 140/90 mmHg without anti-hypertensive treatment (“untreated HT”), while 333 (8.0%) had systolic/diastolic BP ≥ 140/90 mmHg under anti-hypertensive treatment (“uncontrolled HT”) and 300 (7.2%) had systolic/diastolic BP < 140/90 mmHg under anti-hypertensive treatment (“controlled HT”). Consequently, among treated participants, 300 (47.3%) had “controlled HT” while 333 (52.6%) had “uncontrolled HT”. Mean eGFR was 85.9 ± 14.6 ml/min/1.73 m^2^ and mean ACR 4.8 (3.3–8.4) mg/g. The mean follow-up time of included participants was 10.8 ± 0.4 years. The mean KFD was -0.49 ± 1.08 ml/min/1.73 m^2^ per year. A total of 2,912 (70%) participants had a decline in eGFR during the follow-up. The distribution of KFD is presented in Supplementary Figure 1. Table 1 summarizes baseline characteristics of the subjects according to age tertiles. With increasing age tertiles, participants were less frequently men, had higher BMI, higher prevalence of HT, anti-hypertensive drug use, diabetes, and dyslipidaemia but were less frequently smokers. Older participants also had lower eGFR and a higher prevalence of CKD and albuminuria. The incidence of CKD during follow-up and the prevalence of rapid KFD were higher in older participants while the annual KFD showed non-linearity across age tertiles.

**TABLE 1:** Baseline characteristics of included participants according to age tertiles (*n* = 4,163).

	Young (*n* = 1,396)	Middle age (*n* = 1,386)	Old (*n* = 1,381)	*P*-value
**Demographic characteristics**				
Age (years)	40.6 (34.9–46.0)	51.3 (46.1–57.7)	63.6 (57.8–75.4)	**<0.001**
Gender (men)	686 (49.2%)	618 (44.6%)	558 (40.4%)	**<0.001**
Education level - High - Medium - Low	365 (26.2%) 385 (27.6%) 646 (46.3%)	297 (21.4%) 354 (25.5%) 735 (53.0%)	191 (13.8%) 339 (24.6%) 851 (61.6%)	**<0.001**
**Cardiovascular risk factors**				
BMI (kg/m^2^)	24.7 ± 4.1	25.4 ± 4.4	26.3 ± 4.3	**<0.001**
Hypertension^a^	182 (13.0%)	385 (27.8%)	760 (55.0%)	**<0.001**
Office BP ≥ 140/90 mmHg	154 (11.0%)	301 (21.7%)	572 (41.4%)	**<0.001**
Anti-hypertensive drug	54 (3.8%)	169 (12.1%)	410 (29.8%)	**<0.001**
Diabetes	16 (1.1%)	59 (4.3%)	134 (9.7%)	**<0.001**
Dyslipidaemia	25 (1.8%)	104 (7.5%)	283 (20.5%)	**<0.001**
Smoking	411 (29.4%)	366 (26.4%)	256 (18.5%)	**<0.001**
**Anti-hypertensive medication**				
ACE/ARB	33 (2.3%)	123 (8.8%)	309 (22.3%)	**<0.001**
CCB	7 (0.5%)	32 (2.3%)	99 (7.1%)	**<0.001**
Diuretic	12 (0.8%)	55 (3.9%)	145 (10.5%)	**<0.001**
BB	23 (1.6%)	61 (4.4%)	167 (12.0%)	**<0.001**
Other	12 (0.8%)	15 (1.0%)	53 (3.8%)	**<0.001**
**Laboratory variables**				
Creatinine (umol/L)	79.1 ± 14.1	80.3 ± 22.1	81.1 ± 28.5	0.09
eGFR (ml/min/1.73 m^2^)	94.0 ± 13.3	86.4 ± 12.8	77.2 ± 12.7	**<0.001**
Prevalent CKD	8 (0.6%)	30 (2.2%)	118 (8.5%)	**<0.001**
Incident CKD	9 (0.6%)	57 (4.1%)	236 (17.1%)	**<0.001**
KFD (ml/min/1.73 m^2^/year)	−0.43 ± 1.03	−0.39 ± 1.05	−0.66 ± 1.15	**<0.001**
Rapid KFD	13 (0.9%)	20 (1.4%)	48 (3.5%)	**<0.001**
Uric acid (umol/L)	295 ± 79	304 ± 82	324 ± 83	**<0.001**
CRP (mg/L)	0.9 (0.4–2.1)	1.1 (0.6–2.3)	1.6 (0.8–3.2)	**<0.001**
ACR (mg/g)	4.3 (3.1–7.1)	4.8 (3.3–8.0)	5.8 (3.8–10.7)	**<0.001**
ACR ≥ 30 mg/g	56 (4.1%)	61 (4.5%)	97 (7.2%)	**0.001**

BMI, body mass index; BP, blood pressure; eGFR, estimated glomerular filtration rate; CKD, chronic kidney disease; KFD, kidney function decline; CRP, C-reactive protein; ACR, albumin to creatinine ratio.

^a^Defined as the use of anti-hypertensive drug or a mean systolic/diastolic BP ≥ 140/90 mmHg on office measurement. Bold values indicate *p* < 0.05.

### Effect of age on kidney function decline

The global effect of age on the annual KFD is depicted in Supplementary Figure 2. In addition to age (Table 1), many covariates were significantly associated with KFD in univariate and age-adjusted analyses (Supplementary Table 2). As such, these covariates were sequentially included in multivariate models 1, 2, and 3. Results from univariate as well as multivariate linear regression using tertiles of age as a predictor of KFD are presented in Supplementary Table 3. Significant KFD was shown in all three tertiles of age in every model (individual *p*-values given in Supplementary Table 3). Moreover, KFD was significantly different across tertiles of age in every model (*F* test *p* < 0.001). As such, KFD was significantly more pronounced when comparing older to younger participants. In the fully adjusted model (model 3), KFD for the older participants was −0.60 (−0.85 to −0.34) compared to −0.39 (−0.64 to−0.14) ml/min/1.73 m^2^ per year in the youngest. Overall, an increase in one log of ACR was associated with an increased in KDF of −0.11 (−0.15 to −0.76) ml/min/1.73 m^2^ per year.

### Effect of hypertension on kidney function decline related to age

**FIGURE 2:**
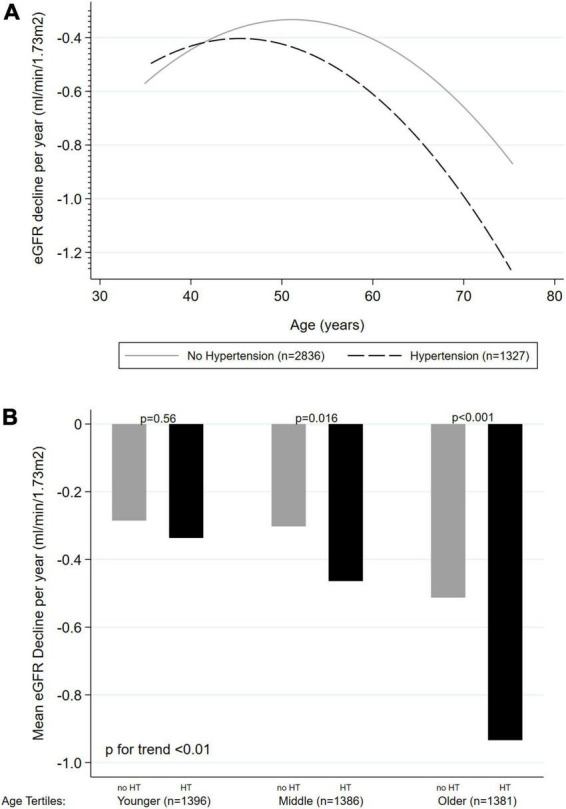
Unadjusted effect of age on kidney function decline (ml/min/1.73 m^2^ per year) according to hypertension status (*n* = 4,163). **(A)** Age as a continuous variable, with quadratic fitted line. **(B)** Age as tertiles categories. KFD, kidney function decline; HT, hypertension.

In all the above-presented models, a significant interaction was found between age and HT on KFD prediction (*p* < 0.001 for LRT). Therefore, analyses were stratified based on the presence or absence of HT (Table 2). The unadjusted effect of age on KFD according to HT status is presented in Figures 2A,B. Significant KFD was shown individually in all three tertiles of age in every model, independently of HT status (individual *p*-values given in Table 2). When considering HT participants, KFD was significantly different across tertiles of age in every model (*F* test *p* < 0.001) with old participants having more pronounced KFD compared to young and middle age participants. When considering non-HT participants only, KFD was not significantly different across tertiles of age (F test *p* = 0.054 in model 3), and borderline in model 2 not accounting for ACR (*F* test *p* = 0.049). In the fully adjusted model (model 3), KFD for older participants was −0.82 (−1.12 to −0.52) ml/min/1.73 m^2^ and −0.45 (−0.71 to −0.19) ml/min/1.73 m^2^ for HT and non-HT participants respectively. The difference between those with HT or without was no so pronounced in younger categories.

**TABLE 2:** Linear regression using tertiles of age as a predictor of kidney function decline stratified according to the presence of hypertension (*n* = 4,163).

	Unadjusted (*n* = 4,163)		Model 1 (*n* = 4,163)		Model 2 (*n* = 4,163)		Model 3 (*n* = 4,040)	
	β (95% CI)	*P*-value	β (95% CI)	*P*-value	β (95% CI)	*P*-value	β (95% CI)	*P*-value
**Young (*n* = 1,396)**								
**HT** (*n* = 182)	−0.45 (−0.60; −0.29)^a^	**<0.001**	−0.87 (−1.10; −0.65)^a^	**<0.001**	−0.58 (−0.87; −0.28)^a^	**<0.001**	−0.32 (−0.63; −0.01)^a^	**0.045**
**No HT** (*n* = 1,214)	−0.43 (−0.49; −0.38)^b^	**<0.001**	−0.85 (−1.01; −0.70)^b^	**<0.001**	−0.59 (−0.82; −0.35)^c^	**<0.001**	−0.37 (−0.62; −0.12)^b^	**0.004**
**Middle age (*n* = 1,396)**								
**HT** (*n* = 385)	−0.43 (−0.62; −0.23)^a^	**<0.001**	−0.85 (−1.10; −0.61)^a^	**<0.001**	−0.59 (−0.88; −0.29)^a^	**<0.001**	−0.36 (−0.67; −0.05)^a^	**0.023**
**No HT** (*n* = 1,001)	−0.37 (−0.44; −0.31)^b^	**<0.001**	−0.80 (−0.96; −0.64)^b^	**<0.001**	−0.55 (−0.78; −0.31)^c^	**<0.001**	−0.32 (−0.57; −0.07)^b^	**0.013**
**Old (*n* = 1,381)**								
**HT** (*n* = 760)	−0.79 (−0.97; −0.60)^a^	**<0.001**	−1.21 (−1.45; −0.98)^a^	**<0.001**	−1.02 (−1.31; −0.74)^a^	**<0.001**	−0.82 (−1.12; −0.52)^a^	**<0.001**
**NoHT** (*n* = 621)	−0.48 (−0.56; −0.39)^b^	**<0.001**	−0.92 (−1.10; −0.74)^b^	**<0.001**	−0.68 (−0.93; −0.43)^c^	**<0.001**	−0.45 (−0.71; −0.19)^b^	**0.001**

KFD, kidney function decline; HT, hypertension.

β coefficients, 95% CI, and associated p-values correspond to the absolute effect of each individual age tertiles on KFD.

Model 1: Adjusted for gender, education level, dyslipidaemia, CRP, uric acid, and interaction term HT × age tertiles.

Model 2: Adjusted as model 1 with the addition of diabetes, BMI, CKD.

Model 3: Adjusted as model 2 with the addition of ACR.

^a^p < 0.001 for difference across tertiles of age. ^b^p = NS for difference across tertiles of age. ^c^p = 0.049 for difference across tertiles of age. Bold values indicate p < 0.05.

In sensitivity analyses, HT definition was modified to include only patients with systolic/diastolic BP ≥ 140/90 mmHg independent of treatment status. Results were qualitatively identical with a significant interaction between age and HT on KFD prediction (*p* < 0.001 for LRT). Analyses were also repeated adjusting for angiotensin-converting enzyme inhibitor (ACE) or angiotensin receptor blocker (ARB), calcium-channel blocker and diuretic medication. Results were qualitatively identical with a significant interaction between age and HT on KFD prediction (*p* < 0.001 for LRT).

### Effect of age and hypertension on rapid kidney function decline and incident chronic kidney disease

In the whole cohort (*n* = 4,163), only 81 (1.9%) had rapid KFD with an increased incidence in older subjects (Table 1, *p* < 0.001). In patients without CKD at baseline (*n* = 4,007), 302 (7.5%) developed new CKD during follow-up. Results from univariate as well as multivariate logistic regression using tertiles of age as a predictor of rapid KFD or incident CKD are presented in Table 3. Log-linearity was confirmed with linear increase in OR across tertiles of age with respect to both rapid KFD and incident CKD. Thus, results are presented as relative increments across tertiles of age rather than individual effect. An increase in tertiles of age was significantly associated with rapid KFD or incident CKD in every model. In the fully adjusted model (model 3), the OR of increasing tertiles of age for rapid KFD and incident CKD prediction was 1.63 (1.17–2.29) and 4.51 (3.54–5.75), respectively. No significant interaction existed between age and HT in the presented models (*p* > 0.5 for LRT).

**TABLE 3:** Logistic regression using tertiles of age as a predictor of rapid kidney function decline or incident chronic kidney disease.

	Unadjusted		Model 1		Model 2		Model 3	
	OR (95% CI)	*P*-value	OR (95% CI)	*P*-value	OR (95% CI)	*P*-value	OR (95% CI)	*P*-value
**Rapid KFD** (*n* = 4,163)								
Increasing tertiles of age	2.05 (1.52; 2.78)	**<0.001**	1.82 (1.33; 2.51)	**<0.001**	1.65 (1.18; 2.31)	**0.003**	1.63 (1.17; 2.29)	**0.004**
**Incident CKD** (*n* = 4,007)								
Increasing tertiles of age	5.55 (4.40; 7.00)	**<0.001**	4.97 (3.92; 6.29)	**<0.001**	4.70 (3.69; 5.99)	**<0.001**	4.51 (3.54; 5.75)	**<0.001**

KFD, kidney function decline; CKD, chronic kidney disease.

Model 1: Adjusted for gender, education level, dyslipidaemia, CRP, and uric acid.

Model 2: Adjusted as model 1 with the addition of HT, diabetes, and BMI.

Model 3: Adjusted as model 2 with the addition of ACR (n = 3,889 with available ACR data). Bold values indicate p < 0.05.

## Discussion

In this population-based study including Caucasian participants aged 35–75, we observed a decline in eGFR in 70% of subjects over a 10 year follow-up with a mean KFD of 0.49 ml/min/1.73 m^2^ per year. After accounting for numerous confounders, age was strongly and non-linearly associated with KFD with a faster decline in older individuals. Thus, KFD in older participants was 0.60 ml/min/1.73 m^2^ per year as compared to 0.33 ml/min/1.73 m^2^ per year in middle-aged patients. This effect was however, mainly driven by the presence of HT as normotensive participants had similar rates of KFD across age groups. Nevertheless, aging was overall associated with a linear increase in the risk of incident CKD, regardless of the presence of HT.

It has long been established that kidney function decreases with time as a consequence of physiological aging (7, 8). While a decline in eGFR of around 1 ml/min/1.73 m^2^ per year starting at around 40 years of age is generally reported, substantial differences in the rate of KFD exist in the literature (31). In 1985, Lindeman et al. reported a decline of 0.75 ml/min per year in eGFR using 24 h creatinine clearance in 254 healthy subjects with a rather long follow-up period (8). Since then, few longitudinal studies reported on the age-associated KFD in large population-based cohorts. Studies focusing exclusively on elderly participants over 65 observed faster rate of eGFR decline with KFD of 2.37 and 2.7 ml/min/1.73 m^2^ per year in Brazilian and Italian cohorts respectively, (12, 13). In a Canadian population, observed KFD ranged from 0.8 to 2.7 ml/min/1.73 m^2^ per year depending on gender as well as the presence of diabetes (11). Other available data are derived from cross-sectional studies or observational extension of clinical trials in patients with CKD (32–34). In our study, the mean KFD was 0.49 ml/min/1.73 m^2^ per year, a rather slow decline in comparison to those prior studies. The main contributor to this discordance is likely represented by the population sample. Compared to previous studies we enrolled a relatively young and healthy population with a low prevalence of risk factors of CKD progression such as HT, diabetes, obesity, and smoking (18, 35–37). Moreover, healthier patients might have been further selected owing to the long follow-up of our study, as excluded patients were globally sicker. At last, Cockcroft-Gault and MDRD eGFR equations were often used in prior studies. In contrast, we used CKD-EPI equation thus potentially resulting in less bias, in particular in a Caucasian population with a low prevalence of CKD (38).

The influence of age on the rate of KFD is a matter of debate. In their seminal study, Lindeman et al. reported that the rate of KFD became faster with aging (8). A Japanese study estimated the rate of KFD over a 10 year follow-up in 120,000 individuals from the general population using MDRD equation (39). While initial kidney function was associated with the subsequent rate of KFD in this study, authors could not detect an independent effect of age as the average KFD was 0.36 ml/min/1.73 m^2^ per year and similar across age groups from 40 to 79 years old. Similarly, in another Japanese study including 45,000 healthy subjects without HT, diabetes or proteinuria, age did not have an influence on the rate of KFD (15). A more recent study from Israel enrolled more than 2,500 healthy participants from the general population and reported a KFD of 0.97 ml/min/1.73 m^2^ per year using CKD-EPI equation over a 5 year follow-up (14). They also noted that KFD significantly increased with aging from 0.82 to 1.15 ml/min/1.73 m^2^ per year in the lowest (20–30 years old) compared to the highest (>50 years old) age group respectively. Those results were, however, not adjusted for potential confounders and albuminuria was notably not included in these analyses. However, albuminuria has consistently been associated with a steeper decline in kidney function in large observational studies (16, 17, 40, 41). We confirmed this association in our longitudinal cohort, as albuminuria was associated both with aging and decline in kidney function. Thus, we could show that albuminuria partially confounded the relationship between aging and subsequent decline in kidney function as accounting for ACR markedly attenuated the effect of age on KFD in multivariate analysis. Overall, an increase in ACR significantly accentuated KFD. The independent effect of age on KFD was, however, still highly significant after adjustment for potential confounders in our cohort. Moreover, this relationship was found to be non-linear with old and middle-aged participants having faster and slower rates of KFD respectively as compared to young participants.

Importantly, we found that the influence of age on the rate of KFD was highly dependent on the presence of HT. In fact, the age effect was blunted in normotensive participants to the point where differences in KFD across age groups could not be detected. In contrast, the presence of HT magnified the effect of age as old hypertensive participants had much faster rates of KFD compared to young and middle aged participants. This phenomenon held true when considering only patients with systolic/diastolic BP ≥ 140/90 mmHg independent of treatment status or when accounting for potential nephroprotection from ACE/ARB medication. In this regard, while a physiological decline of kidney function with age is expected in most individuals, the presence of HT seems to act as an additional burden on its functional reserve, thus substantially accelerating this process. Those findings could point toward a role for optimal HT management as a preventive measure for CKD development in older people. Such a conclusion can, however, not be drawn from observational data. A detrimental effect of HT on eGFR decline has been previously reported in similar studies but interaction analyses were generally not conducted and the intricate interplay between age and HT on the rate of KFD has not been previously described (18, 19, 42). Eriksen et al. found that HT was paradoxically associated with slower eGFR decline in 1,600 participants without diabetes of CKD at baseline (30). Biases inherent to observational studies are, however, likely as this association was specific to the sub-group of patients taking antihypertensive medications and the effect of age was not described in this study. In their Israeli cohort, Cohen et al. did not find an influence of HT on the rate of KFD, but less than 4% of participants had HT (14). Finally, beyond considerations related to the presence of HT, age itself was identified as a strong risk factor for the development of incident CKD as well as rapid decline in kidney function in our cohort. This risk linearly increased with age independently of other predictors of KFD and was not modified by the presence of HT. Globally, aging, HT, and CKD can be perceived as interactive risk factors potentially culminating in clinical frailty, a multidimensional condition associated with susceptibility to stressors and a high risk of death and hospitalization (43). Hypertensive elderly patients are at particularly high risk of frailty and susceptible to physical as well as cognitive decline that could significantly impact their quality of life (44, 45).

Beyond specificities inherent to observational studies, limitations must be taken into account when interpreting our findings. First, a selection bias is possible as excluded participants who did not complete the follow-up were generally sicker than included subjects. This phenomenon is, however, inevitable in large longitudinal cohort studies. Second, kidney function was estimated and not measured. It seems, however, unrealistic to measure GFR in such epidemiological study owing to logistical reasons. Moreover, we used the creatinine-based CKD-EPI equation that has been developed and validated in a population very similar to our study sample. In measuring KFD, we used two time-points and inferred a linear decline over time. While measurement of several time-points would have allowed considering non-linear trajectories, it has recently been shown that eGFR decline could be considered linear over time in non-diabetic as well as diabetic patients (46). Finally, our cohort included Caucasian participants only by design. Potential race discrepancies could therefore not been explored and whether our findings could be extrapolated to other ethnicities is unknown.

## Conclusion

A physiological decline in kidney function over time is present in the majority of people from the general population. Age contributes non-linearly to the rate of this decline with older subjects declining the fastest, independently of common risk factors. The presence of HT is, however, a major facilitating factor in this setting as KFD worsened with age only in hypertensive participants. As such, HT can be viewed as a major pathological factor aggravating the age-related physiological decrease in eGFR in the general population. Those findings highlight the importance of HT as a contributor to CKD in aging.

## Data availability statement

The raw data supporting the conclusions of this article will be made available by the authors, without undue reservation.

## Ethics statement

The study involving human participants was reviewed and approved by the local ethics committee “Commission cantonale d’éthique de la recherche sur l’être humain” (CER-VD: VD-16/03; VD-33/09, and VD-26/14) and was conducted in accordance with the declaration of Helsinki. The patients/participants provided their written informed consent to participate in this study.

## Author contributions

DJ analyzed the data, interpreted the results, and wrote the manuscript. PV collected the data and reviewed the manuscript. MB designed the study and reviewed the manuscript. BP designed the study, analyzed the data, interpreted the results, and reviewed the manuscript. All authors have approved the final version of the manuscript.

## References

[1] Go AS Chertow GM Fan D McCulloch CE Hsu C. Chronic kidney disease and the risks of death, cardiovascular events, and hospitalization. *N Engl J Med.* (2004) 351:1296–305. 10.1056/nejmoa041031 15385656

[2] Wilson S Mone P Jankauskas SS Gambardella J Santulli G. Chronic kidney disease: definition, updated epidemiology, staging, and mechanisms of increased cardiovascular risk. *J Clin Hypertens.* (2021) 23:831–4. 10.1111/jch.14186 33455061 PMC8035205

[3] Brück K Stel VS Gambaro G Hallan S Völzke H Ärnlöv J CKD prevalence varies across the European general population. *J Am Soc Nephrol.* (2016) 27:2135–47. 10.1681/ASN.2015050542 26701975 PMC4926978

[4] Forni Ogna V Ogna A Ponte B Gabutti L Binet I Conen D Prevalence and determinants of chronic kidney disease in the Swiss population. *Swiss Med Wkly.* (2016) 146:w14313. 10.4414/smw.2016.14313 27152492

[5] Schaeffner ES Ebert N Delanaye P Frei U Gaedeke J Jakob O Two novel equations to estimate kidney function in persons aged 70 years or older. *Ann Intern Med.* (2012) 157:471–81. 10.7326/0003-4819-157-7-201210020-00003 23027318

[6] Davies DF Shock NW. Age changes in glomerular filtration rate, effective renal plasma flow, and tubular excretory capacity in adult males. *J Clin Invest.* (1950) 29:496–507. 10.1172/JCI102286 15415454 PMC436086

[7] Rowe JW Andres R Tobin JD Norris AH Shock NW. The effect of age on creatinine clearance in men: a cross sectional and longitudinal study. *J Gerontol.* (1976) 31:155–63. 10.1093/geronj/31.2.155 1249404

[8] Lindeman RD Tobin J Shock NW. Longitudinal studies on the rate of decline in renal function with age. *J Am Geriatr Soc.* (1985) 33:278–85. 10.1111/j.1532-5415.1985.tb07117.x 3989190

[9] Chung SM Lee DJ Hand A Young P Vaidyanathan J Sahajwalla C. Kidney function changes with aging in adults: comparison between cross-sectional and longitudinal data analyses in renal function assessment. *Biopharm Drug Dispos.* (2015) 36:613–21. 10.1002/bdd.1988 26301459

[10] Muntner P. Longitudinal measurements of renal function. *Semin Nephrol.* (2009) 29:650–7. 10.1016/j.semnephrol.2009.07.010 20006797

[11] Hemmelgarn BR Zhang J Manns BJ Tonelli M Larsen E Ghali WA Progression of kidney dysfunction in the community-dwelling elderly. *Kidney Int.* (2006) 69:2155–61. 10.1038/sj.ki.5000270 16531986

[12] Sesso R Prado F Vicioso B Ramos LR. Prospective study of progression of kidney dysfunction in community-dwelling older adults. *Nephrology.* (2008) 13:99–103. 10.1111/j.1440-1797.2008.00919.x 18275496

[13] Giannelli SV Graf CE Herrmann FR Michel JP Patel KV Pizzarelli F Natural history of older adults with impaired kidney function: the InCHIANTI study. *Rejuvenation Res.* (2011) 14:513–23. 10.1089/rej.2011.1179 21954982 PMC3198123

[14] Cohen E Nardi Y Krause I Goldberg E Milo G Garty M A longitudinal assessment of the natural rate of decline in renal function with age. *J Nephrol.* (2014) 27:635–41. 10.1007/s40620-014-0077-9 24643437

[15] Baba M Shimbo T Horio M Ando M Yasuda Y Komatsu Y Longitudinal study of the decline in renal function in healthy subjects. *PLoS One.* (2015) 10:e129036. 10.1371/journal.pone.0129036 26061083 PMC4464887

[16] Halbesma N Kuiken DS Brantsma AH Bakker SJL Wetzels JFM De Zeeuw D Macroalbuminuria is a better risk marker than low estimated GFR to identify individuals at risk for accelerated GFR loss in population screening. *J Am Soc Nephrol.* (2006) 17:2582–90. 10.1681/ASN.2005121352 16899519

[17] Turin TC James M Ravani P Tonelli M Manns BJ Quinn R Proteinuria and rate of change in kidney function in a community-based population. *J Am Soc Nephrol.* (2013) 24:1661–7. 10.1681/ASN.2012111118 23833255 PMC3785273

[18] Fox CS Larson MG Leip EP Culleton B Wilson PWF Levy D. Predictors of new-onset kidney disease in a community-based population. *J Am Med Assoc.* (2004) 291:844–50. 10.1001/jama.291.7.844 14970063

[19] Zoppini G Targher G Chonchol M Ortalda V Negri C Stoico V Predictors of estimated GFR decline in patients with type 2 diabetes and preserved kidney function. *Clin J Am Soc Nephrol.* (2012) 7:401–8. 10.2215/CJN.07650711 22282481

[20] Yun HR Kim H Park JT Chang TI Yoo TH Kang SW Obesity, metabolic abnormality, and progression of CKD. *Am J Kidney Dis.* (2018) 72:400–10. 10.1053/j.ajkd.2018.02.362 29728317

[21] Turin TC Coresh J Tonelli M Stevens PE De Jong PE Farmer CKT Short-term change in kidney function and risk of end-stage renal disease. *Nephrol Dial Transplant.* (2012) 27:3835–43. 10.1093/ndt/gfs263 22764191

[22] Turin TC Coresh J Tonelli M Stevens PE De Jong PE Farmer CKT Change in the estimated glomerular filtration rate over time and risk of all-cause mortality. *Kidney Int.* (2013) 83:684–91. 10.1038/ki.2012.443 23344477

[23] Levey AS Inker LA Matsushita K Greene T Willis K Lewis E GFR decline as an end point for clinical trials in CKD: a scientific workshop sponsored by the national kidney foundation and the US food and drug administration. *Am J Kidney Dis.* (2014) 64:821–35. 10.1053/j.ajkd.2014.07.030 25441437

[24] Firmann M Mayor V Vidal PM Bochud M Pécoud A Hayoz D The CoLaus study: a population-based study to investigate the epidemiology and genetic determinants of cardiovascular risk factors and metabolic syndrome. *BMC Cardiovasc Disord.* (2008) 8:6. 10.1186/1471-2261-8-6 18366642 PMC2311269

[25] Ponte B Pruijm M Marques-Vidal P Martin PY Burnier M Paccaud F Determinants and burden of chronic kidney disease in the population-based CoLaus study: a cross-sectional analysis. *Nephrol Dial Transplant.* (2013) 28:2329–39. 10.1093/ndt/gft206 23825103

[26] Levey AS Stevens LA Schmid CH Zhang Y Castro AF Feldman HI A new equation to estimate glomerular filtration rate. *Ann Intern Med.* (2009) 150:604–12. 10.7326/0003-4819-150-9-200905050-00006 19414839 PMC2763564

[27] Rifkin DE Shlipak MG Katz R Fried LF Siscovick D Chonchol M Rapid kidney function decline and mortality risk in older adults. *Arch Intern Med.* (2008) 168:2212–8. 10.1001/archinte.168.20.2212 19001197 PMC2879064

[28] Neugarten J Acharya A Silbiger SR. Effect of gender on the progression of nondiabetic renal disease: a meta-analysis. *J Am Soc Nephrol.* (2000) 11:319–29. 10.1681/ASN.V112319 10665939

[29] Eriksen BO Ingebretsen OC. The progression of chronic kidney disease: a 10-year population-based study of the effects of gender and age. *Kidney Int.* (2006) 69:375–82. 10.1038/sj.ki.5000058 16408129

[30] Eriksen BO Stefansson VTN Jenssen TG Mathisen UD Schei J Solbu MD Blood pressure and age-related GFR decline in the general population. *BMC Nephrol.* (2017) 18:77. 10.1186/s12882-017-0496-7 28245797 PMC5331738

[31] Musso CG Oreopoulos DG. Aging and physiological changes of the kidneys including changes in glomerular filtration rate. *Nephron Physiol.* (2011) 119(Suppl. 1):1–5. 10.1159/000328010 21832859

[32] Hunsicker LG Adler S Caggiula A England BK Greene T Kusek JW Predictors of the progression of renal disease in the modification of diet in renal disease study. *Kidney Int.* (1997) 51:1908–19. 10.1038/ki.1997.260 9186882

[33] Wetzels JFM Kiemeney LALM Swinkels DW Willems HL den Heijer M. Age- and gender-specific reference values of estimated GFR in Caucasians: the Nijmegen biomedical study. *Kidney Int.* (2007) 72:632–7. 10.1038/sj.ki.5002374 17568781

[34] Appel LJ Wright JT Greene T Kusek JW Lewis JB Wang X Long-term effects of renin-angiotensin system-blocking therapy and a low blood pressure goal on progression of hypertensive chronic kidney disease in African Americans. *Arch Intern Med.* (2008) 168:832–9. 10.1001/archinte.168.8.832 18443258 PMC3870204

[35] Bleyer AJ Shemanski LR Burke GL Hansen KJ Appel RG. Tobacco, hypertension, and vascular disease: risk factors for renal functional decline in an older population. *Kidney Int.* (2000) 57:2072–9. 10.1046/j.1523-1755.2000.00056.x 10792626

[36] de Boer IH Katz R Fried LF Ix JH Luchsinger J Sarnak MJ Obesity and change in estimated GFR among older adults. *Am J Kidney Dis.* (2009) 54:1043–51. 10.1053/j.ajkd.2009.07.018 19782454 PMC2787647

[37] van der Burgh AC Rizopoulos D Ikram MA Hoorn EJ Chaker L. Determinants of the evolution of kidney function with age. *Kidney Int Rep.* (2021) 6:3054–63. 10.1016/j.ekir.2021.10.006 34901574 PMC8640542

[38] Murata K Baumann NA Saenger AK Larson TS Rule AD Lieske JC. Relative performance of the MDRD and CKD-EPI equations for estimating glomerular filtration rate among patients with varied clinical presentations. *Clin J Am Soc Nephrol.* (2011) 6:1963–72. 10.2215/CJN.02300311 21737852 PMC3156428

[39] Imai E Horio M Yamagata K Iseki K Hara S Ura N Slower decline of glomerular filtration rate in the Japanese general population: a longitudinal 10-year follow-up study. *Hypertens Res.* (2008) 31:433–41. 10.1291/hypres.31.433 18497462

[40] Coresh J Turin TC Matsushita K Sang Y Ballew SH Appel LJ Decline in estimated glomerular filtration rate and subsequent risk of end-stage renal disease and mortality. *JAMA J Am Med Assoc.* (2014) 311:2518–31. 10.1001/jama.2014.6634 24892770 PMC4172342

[41] Shankar A Sun L Klein BEK Lee KE Muntner P Nieto FJ Markers of inflammation predict the long-term risk of developing chronic kidney disease: a population-based cohort study. *Kidney Int.* (2011) 80:1231–8. 10.1038/ki.2011.283 21866089 PMC3260339

[42] Polonia J Azevedo A Monte M Silva JA Bertoquini S. Annual deterioration of renal function in hypertensive patients with and without diabetes. *Vasc Health Risk Manag.* (2017) 13:231–7. 10.2147/VHRM.S135253 28721063 PMC5498504

[43] Clegg A Young J Iliffe S Rikkert MO Rockwood K. Frailty in elderly people. *Lancet.* (2013) 381:752–62. 10.1016/S0140-6736(12)62167-9 23395245 PMC4098658

[44] Mone P Gambardella J Lombardi A Pansini A De Gennaro S Leo AL Correlation of physical and cognitive impairment in diabetic and hypertensive frail older adults. *Cardiovasc Diabetol.* (2022) 21:10. 10.1186/s12933-021-01442-z 35045834 PMC8772197

[45] Mone P Pansini A Frullone S de Donato A Buonincontri V De Blasiis P Physical decline and cognitive impairment in frail hypertensive elders during COVID-19. *Eur J Intern Med.* (2022) 99:89–92. 10.1016/j.ejim.2022.03.012 35300886 PMC8919809

[46] Weldegiorgis M de Zeeuw D Li L Parving HH Hou FF Remuzzi G Longitudinal estimated GFR trajectories in patients with and without Type 2 diabetes and nephropathy. *Am J Kidney Dis.* (2018) 71:91–101. 10.1053/j.ajkd.2017.08.010 29153995

