# Supplementary material for: Aging and hypertension in kidney function decline: A 10 year population-based study
Source: Front Cardiovasc Med. 2022 Oct 6;9:1035313. doi: 10.3389/fcvm.2022.1035313 (PMC9582457; doi:10.3389/fcvm.2022.1035313)

AGING AND HYPERTENSION IN KIDNEY FUNCTION DECLINE: A 10-YEAR POPULATION-BASED STUDY.

David A. JAQUES^1^, Peter VOLLENWEIDER^2^, Murielle BOCHUD^3^, Belen PONTE^1,3^.

1: Division of Nephrology, Department of Medicine, Geneva University Hospitals, Geneva, Switzerland

2: Department of Internal Medicine, University Hospital of Lausanne, Lausanne, Switzerland

3: Department of Epidemiology and Health Systems, University Center of General Medicine and Public Health, Lausanne, Switzerland

**Correspondence:**David A. JAQUES; Belen PONTE
[david.jaques@hcuge.ch](mailto:david.jaques@hcuge.ch); belen.ponte@hcuge.ch

**SUPPLEMENTARY MATERIAL**

**Supplementary tables: 3**

**Supplementary figures: 2**

Supplementary Tables

**Supplementary table 1**: Comparison of baseline characteristics between included and excluded participants (n=6’184).

|  | **Included participants (N=4’163)** | **Excluded participants (N=2’021)** | **P value** |
| --- | --- | --- | --- |
| **Demographic characteristics** | | | |
| Age (years) | 52.2 +/- 10.4 | 55.0 +/- 11.2 | **<0.001** |
| Gender (men) | 1’862 (44.7%) | 1’071 (53.0%) | **<0.001** |
| Education level  - High  - Medium  - Low | 853 (20.5%)  1’078 (25.9%)  2’232 (53.6%) | 287 (14.2%)  390 (19.4%)  1’339 (66.4%) | **<0.001** |
| **Cardiovascular risk factors** | | | |
| BMI (kg/m^2^) | 25.5 +/- 4.3 | 26.6 +/- 5.0 | **<0.001** |
| Hypertension | 1’327 (31.9%) | 925 (45.8%) | **<0.001** |
| Diabetes | 209 (5.0%) | 198 (9.9%) | **<0.001** |
| Dyslipidaemia | 412 (9.9%) | 301 (14.9%) | **<0.001** |
| Smoking | 1’033 (24.8%) | 640 (31.7%) | **<0.001** |
| **Laboratory variables** | | | |
| Creatinine (umol/L) | 79.5 +/- 17.4 | 81.1 +/- 28.5 | **0.007** |
| eGFR (mL/min/1.73m^2^) | 85.9 +/- 14.6 | 85.1 +/- 16.4 | 0.08 |
| Prevalent CKD | 156 (3.8%) | 128 (6.3%) | **<0.001** |
| Uric acid (umol/L) | 307.8 +/- 82.6 | 325.0 +/- 87.0 | **<0.001** |
| CRP (mg/L) | 1.2 (0.6 – 2.6) | 1.5 (0.7 – 3.2) | **<0.001** |
| ACR (mg/g) | 4.8 (3.3 – 8.4) | 5.6 (3.7 – 11.1) | **<0.001** |
| ACR ≥30 mg/g | 214 (5.3%) | 166 (8.5%) | **<0.001** |

*Abbreviations: BMI, body mass index; eGFR, estimated glomerular filtration rate; CKD, chronic kidney disease; CRP, C-reactive protein; ACR, albumin to creatinine ratio.*

**Supplementary table 2**: Association of covariates with KFD in univariate and age-adjusted analyses (n=4’163).

|  | **Unadjusted** | | **Age-adjusted** | |
| --- | --- | --- | --- | --- |
|  | **β (95% CI)** | **P value** | **β (95% CI)** | **P value** |
| **Gender (men)** | -0.10 (-0.17; -0.03) | **0.003** | -0.11 (-0.18; -0.05) | **0.001** |
| **Education level ***  - Medium  - Low | 0.00 (-0.10; 0.10)  -0.13 (-0.21; -0.04) | **<0.001**  0.97  **0.004** | 0.02 (-0.08; 0.12)  -0.09 (-0.18; -0.01) | **<0.001**  0.66  **0.034** |
| **BMI (kg/m^2^)** | -0.02 (-0.03; -0.01) | **<0.001** | -0.02 (-0.02; -0.01) | **<0.001** |
| **Hypertension** | -0.23 (-0.30; -0.16) | **<0.001** | -0.17 (-0.25; -0.10) | **<0.001** |
| **Diabetes** | -0.43 (-0.59; -0.29) | **<0.001** | -0.37 (-0.52; -0.22) | **<0.001** |
| **Dyslipidaemia** | -0.23 (-0.34; -0.12) | **<0.001** | -0.15 (-0.26; -0.03) | **0.012** |
| **Smoking** | -0.05 (-0.13; 0.02) | 0.17 | -0.08 (-0.16; 0.00) | **0.039** |
| **Prevalent CKD** | 0.81 (0.64; 0.98) | **<0.001** | 0.95 (0.78; 1.12) | **<0.001** |
| **Uric acid (umol/L) ∞** | 0.04 (0.00; 0.08) | **0.040** | 0.06 (0.02; 0.01) | **0.003** |
| **CRP (umol/L) ∞** | -0.09 (-0.12; -0.06) | **<0.001** | -0.07 (-0.10; -0.04) | **<0.001** |
| **ACR (mg/g) ∞** | -0.14 (-0.18; -0.10) | **<0.001** | -0.12 (-0.16; -0.08) | **<0.001** |

*Abbreviations: KFD, kidney function decline; BMI, body mass index; CKD, chronic kidney disease; CRP, C-reactive protein; ACR, albumin to creatinine ratio.*

*Category of reference= High educational level.

∞ Coefficients correspond to an increase in 100 umol/l for uric acid, and ACR to an increase in 1 log for CRP and ACR.

**Supplementary table 3**: Linear regression using tertiles of age as a predictor of KFD (n=4’163).

|  | **Unadjusted** | | **Model 1** | | **Model 2** | |
| --- | --- | --- | --- | --- | --- | --- |
|  | **β (95% CI)** | **P value** | **β (95% CI)** | **P value** | **β (95% CI)** | **P value** |
| Young (n=1’396) | -0.43 (-0.49; -0.38)^a^ | **<0.001** | -0.81 (-0.96; -0.65) ^a^ | **<0.001** | -0.61 (-0.84; -0.37) ^a^ | **<0.001** |
| Middle age (n=1’386) | -0.39 (-0.45; -0.34) ^a^ | **<0.001** | -0.77 (-0.93; -0.61) ^a^ | **<0.001** | -0.55 (-0.79; -0.31) ^a^ | **<0.001** |
| Old (n=1’381) | -0.66 (-0.71; -0.60) ^a^ | **<0.001** | -1.03 (-1.20; -0.86) ^a^ | **<0.001** | -0.82 (-1.06; -0.57) ^a^ | **<0.001** |
| **Adjusted for ACR in addition to other covariates (n=4’040)** | | | | | | |
| Young (n=1’354) | -0.23 (-0.31; -0.14)^a^ | **<0.001** | -0.15 (-0.24; -0.60) ^a^ | **<0.001** | -0.39 (-0.64; -0.14) ^a^ | **0.002** |
| Middle age (n=1’344) | -0.17 (-0.26; -0.08) ^a^ | **<0.001** | -0.09 (-0.19; -0.00) ^a^ | **0.049** | -0.33 (-0.58; -0.07) ^a^ | **0.011** |
| Old (n=1’342) | -0.42 (-0.52; -0.33) ^a^ | **<0.001** | -0.35 (-0.45; -0.25) ^a^ | **<0.001** | -0.60 (-0.85; -0.34) ^a^ | **<0.001** |

*Abbreviations: KFD, kidney function decline.*

β coefficients, 95% CI and associated p-values correspond to the absolute effect of each individual age tertiles on KFD.

Model 1: Adjusted for gender, education level, dyslipidaemia, CRP and uric acid.

Model 2: Adjusted as model 1 with the addition of HT, diabetes, BMI and CKD.

a: p<0.001 for difference across tertiles of age.

Supplementary Figures

**Supplementary figure 1**: Kernel density distribution of change in eGFR (mL/min/1.73m^2^) per year in participants (n=4’163).

*Abbreviations: eGFR, estimated glomerular filtration rate.*


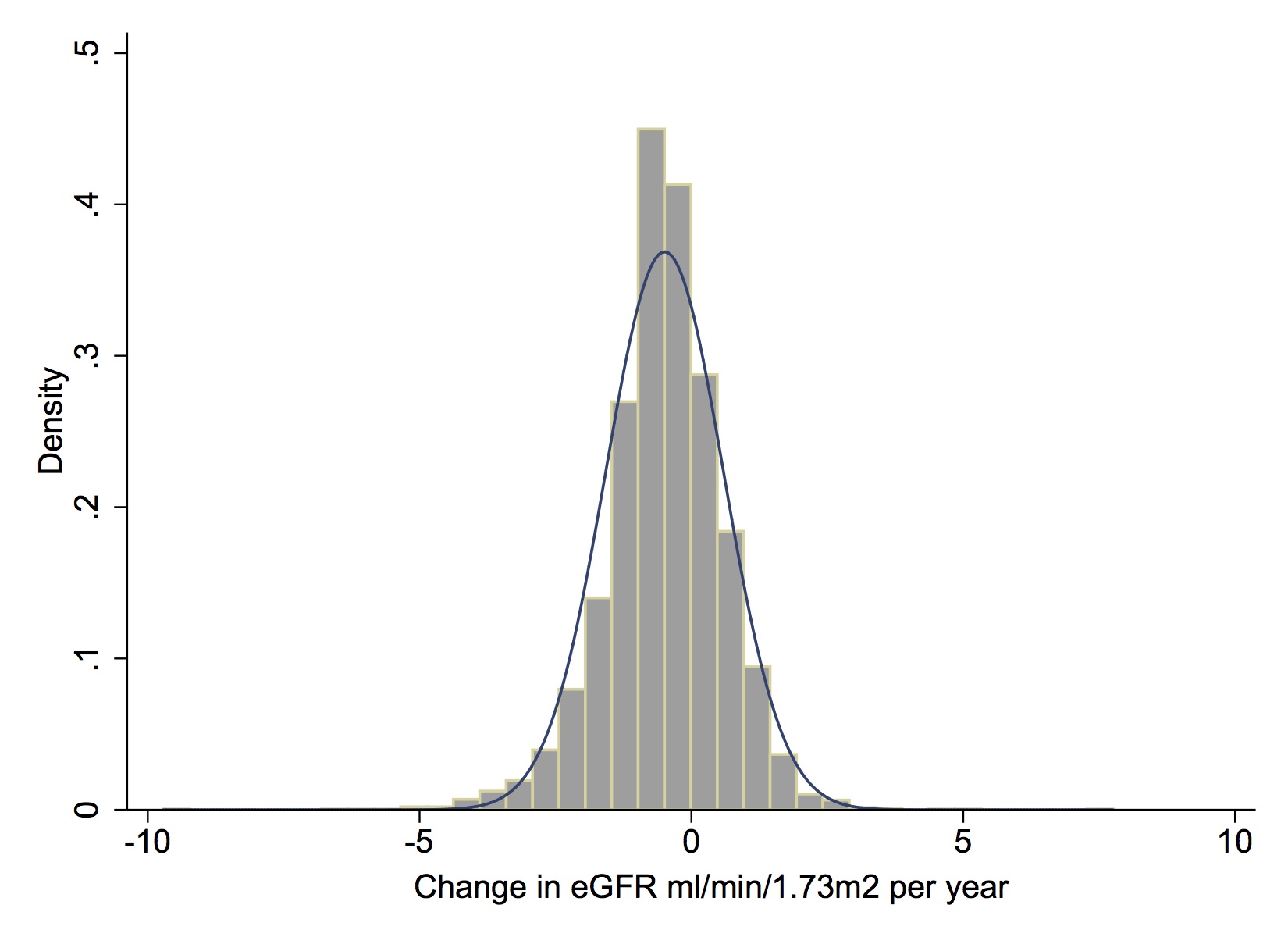


**Supplementary figure 2:** Scatterplot showing the association of age with KFD per year and fitted regression lines, both lowess and linear.

*Abbreviations: KFD, kidney function decline.*


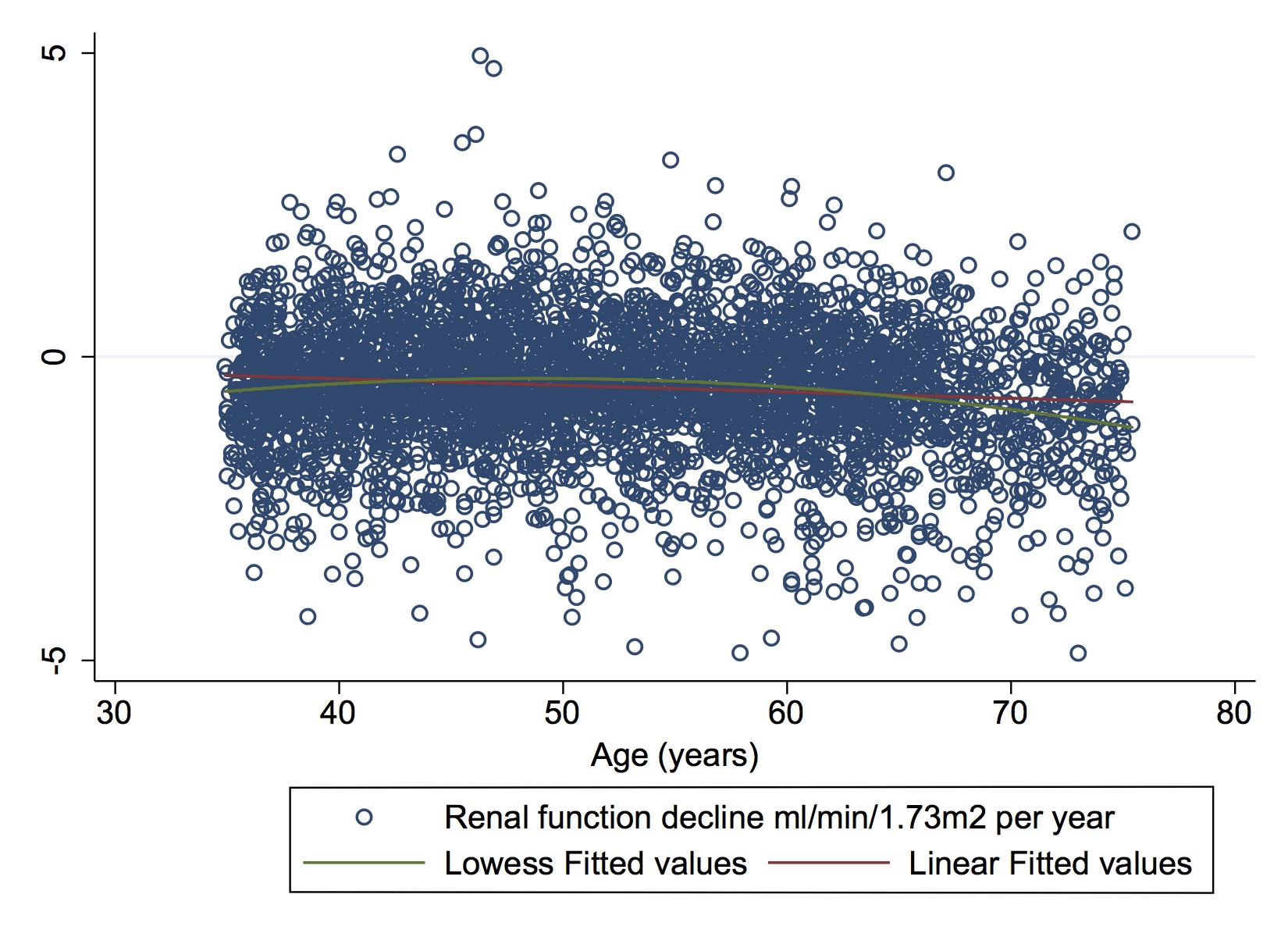

Supplement: Supplementary file 1 [file Data_Sheet_1.DOCX]
